# Supplementary material for: Investigating Tacrolimus Disposition in Paediatric Patients with a Physiologically Based Pharmacokinetic Model Incorporating CYP3A4 Ontogeny, Mechanistic Absorption and Red Blood Cell Binding
Source: Pharmaceutics. 2023 Aug 29;15(9):2231. doi: 10.3390/pharmaceutics15092231 (PMC10536648; doi:10.3390/pharmaceutics15092231)
Supplement: Supplementary file 1 [file pharmaceutics-15-02231-s001.zip › pharmaceutics-2542411-supplementary.pdf]

Supplementary data

Biorelevant media composition

Supplementary Table S1: composition of the used biorelevant media

	Taurocholate (mM)	Lecithin (mM)	Buffer type	pH
Acetate buffer	-	-	Acetate	4.5
SGF	-	-	-	2
½ FaSSIF	1.5	0.375	Phosphate	6.5
FaSSIF	3	0.75	Phosphate	6.5
FeSSIF	15	3.75	Acetate	5

Analytical methods

For the UPLC-UV method, an Acquity UPLC BEH C18 1.7 µm column was used at 60 °C with a flowrate of 0.8 mL/min and injection volume of 80 µL. Tacrolimus was eluted using a mixture of mobile phase A (90% water with 1% trifluoroacetic acid, 10% acetonitrile) and mobile phase B (90% acetonitrile, 10% water) according to the following gradient: an initial A-B ratio of 55-45 changed a ratio of 45-55 over 8 minutes. This ratio was then changed back to 55-45 over 30 seconds which was then maintained for the remaining 3.5 minutes of the HPLC method. For detection, the wavelength was set at 210 nm. The used method was linear between 0.625 and 800 µg/mL.

For the HPLC-MS method, the method validation showed linearity from 31 pM to 1 µM concentrations. A mixture of acetonitrile (mobile phase A) and ammonium acetate 2 mM + 0.1% formic acid (mobile phase B) was used as a mobile phase. After an injection of 5 µL, tacrolimus was eluted at 0.6mL/min using a gradient starting at a 25-75 mobile phase A-B ratio which changed to a 97.5-2.5 A-B ratio over 30 seconds. Next, the composition changed back to a 25-75 ratio over 1.5 minutes. This ratio was then maintained for 1.2 minutes. Separation was achieved using a Kinetex 2.6 µm XB-C18 100A 50x2.1 mm column maintained at 55°C. Detection was done by Electrospray MS-MS using [13C, 2H4]-Tacrolimus as an internal standard. Detection of tacrolimus and its internal standard was performed using 821.55 → 768.53 as a mass transition for tacrolimus-NH4 and 826.600 → 773.57 as a mass transition for its deuterated standard. Electrospray settings were set to the following parameters: capillary voltage: 1.00 kV, cone voltage: 15 V, desolvation temperature: 500°C, desolvation gas flow: 600 L/Hr, cone gas flow: 20 L/h.

CYP3A4 and CYP3A5 ontogeny input data

Supplementary Table S2: Input data for the required ontogeny profiles of the respective enzyme in the respective organ

**Sigmoidal equation**	Emoto liver CYP3A4 ontogeny	Upreti Liver/intestine CYP3A4 ontogeny	Salem liver CYP3A4 ontogeny	Johnson intestine CYP3A4 ontogeny
Fmax	1.78	1.7	1.06	1.059

Fbirth	0.15	0.15	0.11	0.42
Age50	0.18	0.1	0.64	2.357
n	2.42	1.3	1.91	1
Age-cap	0.89	2.5	25	18
**Additional function**				
C0	0.98	0.7	n/a	n/a
C1	1	1	n/a	n/a
Age-cap	25	12.5	n/a	n/a
C2	-0.14	-0.1	n/a	n/a
C3	0.89	0.5	n/a	n/a

Biorelevant solubility data

Supplementary Table S3: Solubility of tacrolimus at each measured data in different biorelevant media

	**Crystalline tacrolimus (µg/mL)**		
	**1h**	**4h**	**24h**
Acetate buffer pH 4.5	0.63 ± 0.75	0.98 ± 0.61	1.65 ± 0.20
SGF	11.58 ± 2.56	10.74 ± 2.33	10.03 ± 0.46
½ FaSSIF	5.41 ± 0.19	5.27 ± 0.55	4.07 ± 0.18
FaSSIF	6.85 ± 0.30	7.51 ± 0.16	6.78 ± 0.29
FeSSIF	17.12 ± 0.17	17.57 ± 0.49	17.46 ± 1.02
	**Tacrolimus amorphous formulation (µg/mL)**		
	**30 min**	**1h**	**4h**
Acetate buffer pH 4.5	57.32 ± 1.74	62.57 ± 1.09	64.53 ± 0.84
SGF	76.69 ± 7.70	74.12 ± 11.38	70.82 ± 1.42
½ FaSSIF	171.14 ± 1.83	174.03 ± 8.97	40.41 ± 1.36
FaSSIF	195.57 ± 6.01	196.558 ± 0.79	67.91 ± 2.77
FeSSIF	583.78 ± 5.96	605.47 ± 18.39	159.34 ± 22.80

Adult model building

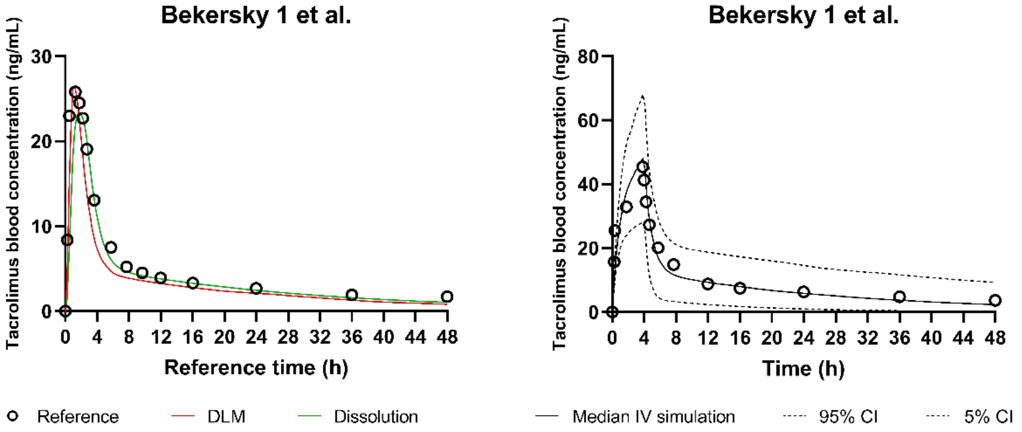

Supplementary Figure S1: Simulation of the reference data by Bekersky et al. used for model development

Adult model performance

IV administration

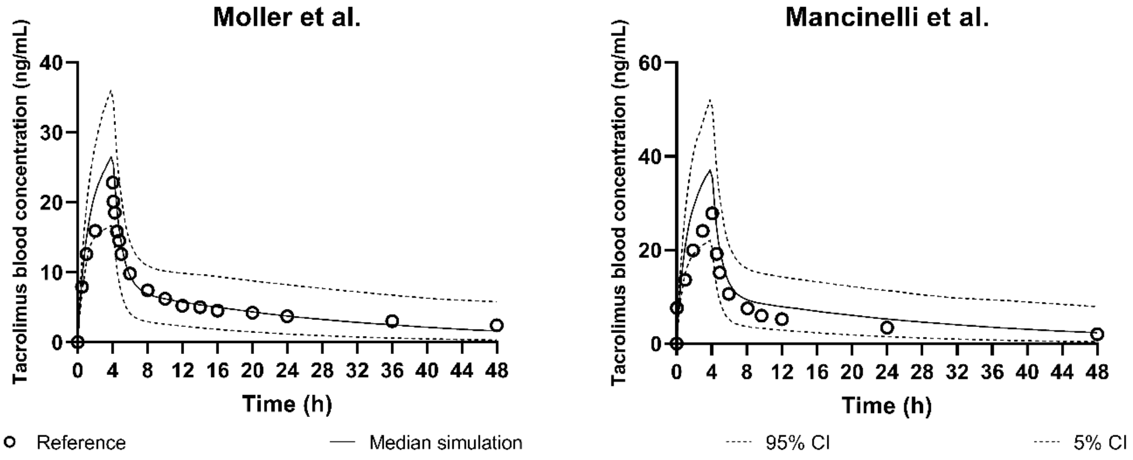

Supplementary Figure S2: Simulation of IV administration to healthy adults of literature reference data

Oral administration

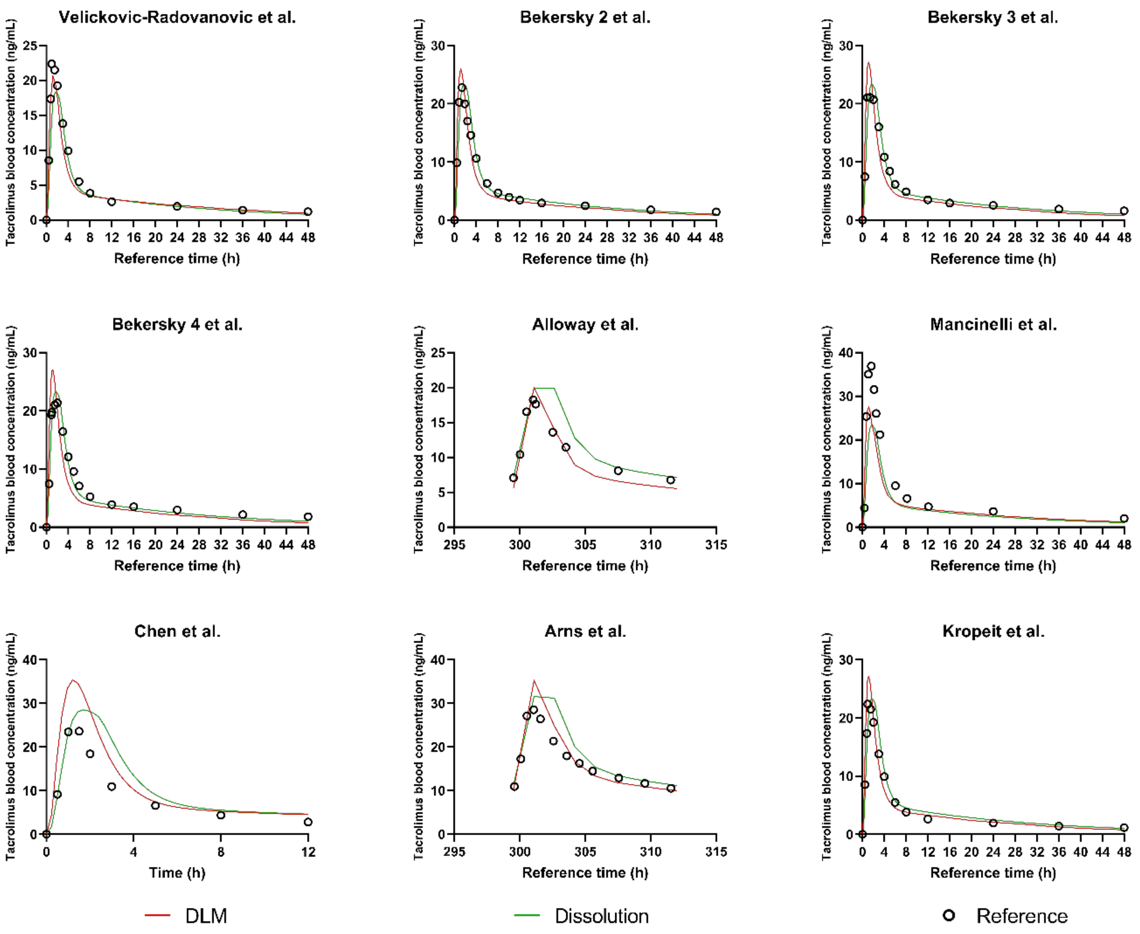

Supplementary Figure S3: Simulations of the literature reference data for oral administration in adults using the different dissolution models. Bekersky 1, 2, 3, 4, Moller and Mancinelli et al. were healthy subjects receiving a single oral dose. Chen and Kropeit et al., were adult kidney transplant patients receiving a single oral dose. Alloway and Arns et al., were adult kidney transplant patients at steady state.

Supplementary Table S4: Model performance for the prediction of different PK parameters using the different dissolution models for the adult population. AFE = Average fold error, AAFE = absolute average fold error

	**IV**			**Oral**	
	**AFE (n=3)**	**AAFE (n=3)**		**AFE (n=9)**	**AAFE (n=9)**
AUC	1.10	1.15	DLM	0.91	1.19
			Dissolution	0.99	1.17
C_{max}	1.18	1.18	DLM	1.13	1.20
			Dissolution	0.99	1.15
k_e	1.16	1.16	DLM	1.19	1.20
			Dissolution	1.04	1.11

Supplementary Table S5: Predicted PK parameters compared to observations for adult reference data

	AUC (ng*h/ml)				Cmax (ng/mL)				Ke (ng/mL/h)			
**Reference**	**Ref**	**DLM**	**Diss.**	**Sol.**	**Ref**	**DLM**	**Diss.**	**Sol.**	**Ref**	**DLM**	**Diss.**	**Sol.**
Alloway et al.	124.94	116.16	145.98	n/a	18.25	20.02	19.93	n/a	0.09	0.12	0.09	n/a
Bekersky 1 et al.	212.26	160.61	183.96	n/a	25.81	26.48	23.34	n/a	0.06	0.07	0.06	n/a
Bekersky 2 et al.	184.83	160.81	183.96	n/a	22.8	25.94	23.34	n/a	0.06	0.07	0.07	n/a
Bekersky 3 et al.	189.67	161.2	183.96	n/a	21.09	27.11	23.34	n/a	0.06	0.08	0.07	n/a
Bekersky 4 et al.	207.64	161.07	183.96	n/a	21.35	27.08	23.34	n/a	0.05	0.08	0.07	n/a
Chen et al.	95.66	131.37	131.07	n/a	23.61	35.34	28.53	n/a	0.2	0.19	0.17	n/a
Kropeit et al.	160.57	161.2	183.96	n/a	22.36	27.11	23.34	n/a	0.06	0.08	0.07	n/a
Mancinelli et al.	281.46	198.16	183.96	n/a	36.91	27.53	23.34	n/a	0.06	0.07	0.07	n/a
Moller et al.	259.13	n/a	n/a	147.12	32.3	n/a	n/a	20.37	0.06	n/a	n/a	0.07
Velikovic-Radanovic et al.	93.52	82.5	84.56	n/a	22.19	21.93	18.28	n/a	0.19	0.19	0.17	n/a
Arns et al.	195.38	208.43	229.76	n/a	28.5	35.23	31.55	n/a	0.09	0.12	0.10	n/a

Paediatric model performance

Effect of absorption

Supplementary Table S6: Paediatric model performance using the different solubilities in biorelevant media (acetate buffer, FaSSIf, ½ FaSSIF) with varying bile salt concentrations as input for the intrinsic solubility (C_0)

		**AUC**	**Cmax (ng/mL)**	**C0 (ng/mL)**	**ke 4-12 (ng/mL/h)**	**Individual timepoints**
Paediatric dissolution	AFE	1.27	1.69	0.89	1.16	1.27
	AAFE	1.40	1.73	1.40	1.34	1.61
	Slope	0.01	-0.05	0.03	-0.02	0.00
DLM<br>C_0 = acetate buffer solubility	AFE	1.10	1.44	0.78	1.18	1.11
	AAFE	1.30	1.52	1.46	1.34	1.51
	Slope	0.01	-0.03	0.03	-0.02	0.01
DLM<br>C_0 = FaSSIF solubility	AFE	1.09	1.43	0.77	1.11	1.12
	AAFE	1.30	1.51	1.46	1.43	1.50
	Slope	0.01	-0.03	0.03	-0.12	0.01
DLM<br>C_0 = ½ FaSSIF solubility	AFE	1.09	1.43	0.77	1.11	1.11
	AAFE	1.30	1.51	1.46	1.43	1.50
	Slope	0.01	-0.03	0.03	-0.12	0.01

Fold errors different paediatric dissolution models and effect of bile salts

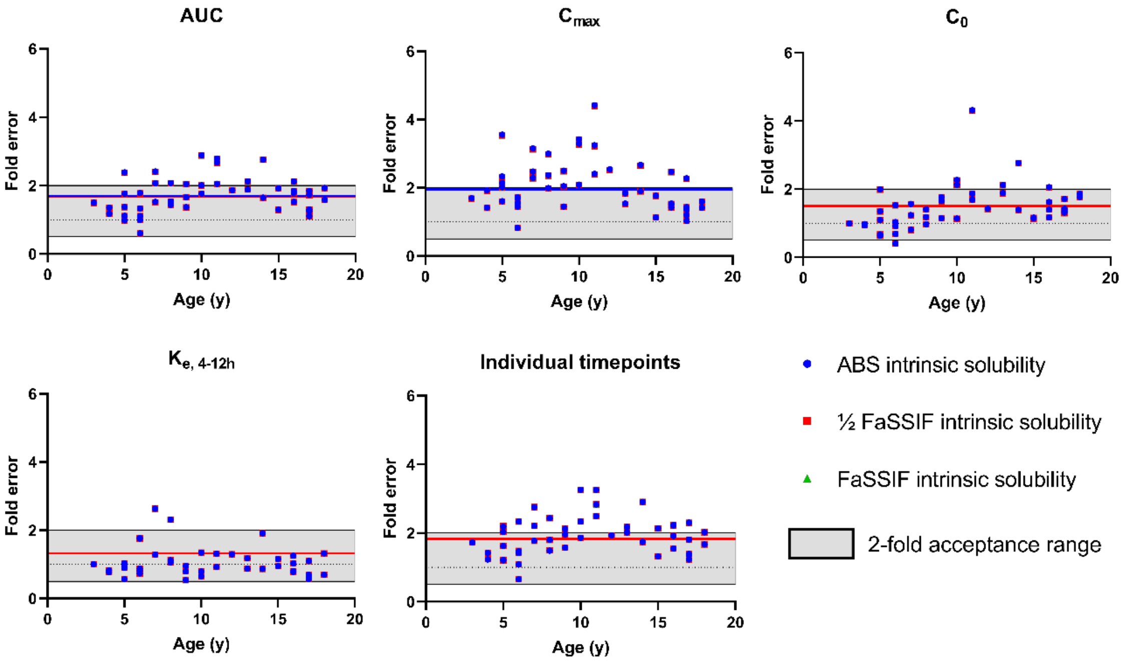

Supplementary Figure S4: Fold error on simulated PK parameters as a function of age. Grey coloured area indicates the 2-fold acceptance criteria. Blue dots = Simulations using the DLM and solubility in acetate buffer pH 4.5 as intrinsic solubility, Red squares = Simulations using the DLM and solubility in half concentrated FaSSIF as intrinsic solubility, Green triangle = Simulations using the DLM and solubility in FaSSIF as intrinsic solubility. Full line indicates the corresponding average fold error. All concentrations are at steady state.
